# P-2056. Impact of social determinants and care access on outcomes for people with HIV at a Ryan White funded clinic

**DOI:** 10.1093/ofid/ofaf695.2220

**Published:** 2026-01-11

**Authors:** Coby L Bessinger, Tiffany Stivers, Alice C Thornton, Armaghan-e-Rehman Mansoor

**Affiliations:** University of Kentucky College of Medicine, New Orleans , LA; University of Kentucky, Lexington, Kentucky; The University of Kentucky, Lexington, Kentucky; University of Kentucky, Lexington, Kentucky

## Abstract

**Background:**

Social and demographic determinants significantly impact outcomes along the human immunodeficiency virus (HIV) care continuum. Viral suppression and achievement of a CD4 T-cell count above threshold for advanced HIV are important goals for the management of HIV. Our study explores the association between social, demographic and behavioral determinants at a clinic receiving Ryan White HIV/AIDS program (RWHAP) funds and providing comprehensive services for people with HIV (PWH) in Southeastern United States.

**Table 1.**
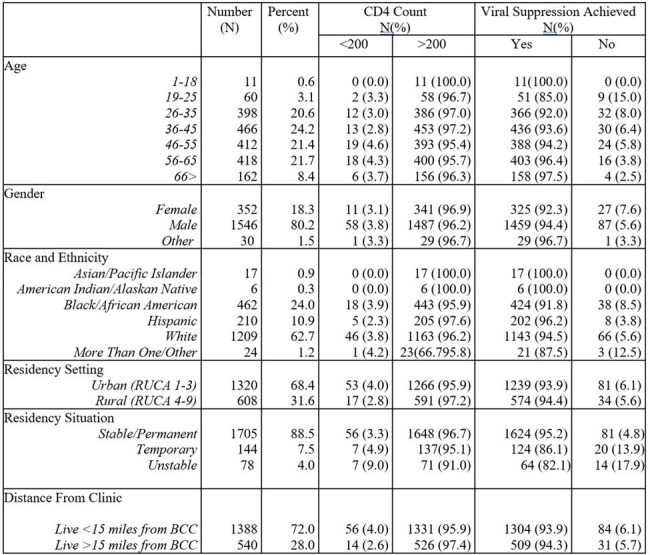
Demographic data for PWH seen at the BCC

**Table 2.**
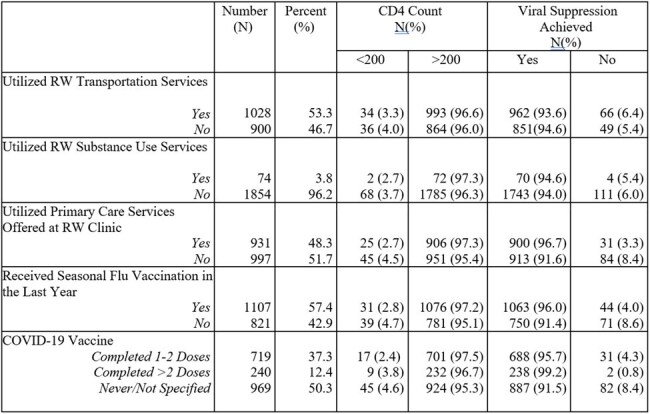
Utilization of RW Services at BCC and Preventative Healthcare

**Methods:**

All PWH actively enrolled in any RW services between January and September 2024 were included. Demographics, social determinants, use of RW services, viral load and CD4 count were obtained through the CAREWare software and supplemented by chart review. A multinomial logistic regression analysis was performed to determine associations between determinants and viral suppression, and proportion of patients with a CD4 count > 200.

**Results:**

A total of 1928 PWH were included, with a median age of 46 (IQR 36-58), of whom 1546 (80.2%) identified as male. Of the cohort, 94% had achieved viral suppression, and 96% had a CD4 >200. 1705 patients (88.5%) had stable housing, and 1388 (72.0%) lived within 15 miles of the clinic location (Table 1). Primary care services at the clinic were utilized by 931 (48.3%). In the multinomial logistic regression, we found a strong association with viral suppression (B= 2.237, 95% CI: 1.426 - 3.508) and CD4 > 200 (B= 1.710, 95% CI: 1.008 – 2.902) and receipt of primary care services, and a protective effect for PWH with stable housing (B= 0.346, 95% CI: 0.176- 0.677). PWH with multiple risk factors for HIV acquisition were at a high risk for not achieving viral suppression (B= 2.273, 95% CI: 1.164- 4.436). 969 individuals (50.3%) had never received a COVID-19 vaccine, which had a strong correlation with unsuppressed viral load (B= 2.003, CI: 95% 1.259-3.185). We did not find an association between distance from clinic, rural vs urban residence or insurance status.

**Conclusion:**

PWH engaged in comprehensive in-house primary care services and preventive healthcare, and for whom housing needs were fulfilled had a significantly higher likelihood of achieving viral suppression and CD4 counts > 200 cells/µL.

**Disclosures:**

All Authors: No reported disclosures

